# Nobel Peace Prize nomination for Doctors Against Forced Organ Harvesting (DAFOH) – a recognition of upholding ethical practices in medicine

**DOI:** 10.3325/cmj.2016.57.219

**Published:** 2016-06

**Authors:** Alan Šućur, Srećko Gajović

**Affiliations:** 1 Department of Physiology and Immunology, University of Zagreb School of Medicine, Zagreb, Croatia *alan.sucur@mef.hr*; 2 Department of Histology and Embryology, Croatian Institute for Brain Research, University of Zagreb School of Medicine, Zagreb, Croatia; Šućur and Gajović: Nobel Peace Prize nomination for DAFOH

“The world is a dangerous place to live; not because of the people who are evil, but because of the people who don't do anything about it.”

Albert Einstein

A mere nomination for the Nobel Peace Prize, the “world’s most prestigious prize,” already attracts significant attention to the nominee, acts as an acknowledgment by itself, and serves as a catalyst for addressing the issue at hand. Although major advances in medicine are annually rewarded with a Nobel Prize, equally important ethical and humanitarian aspects of medicine have received much less attention, with the only two medical organizations awarded the Nobel Peace Prize being International Physicians for the Prevention of Nuclear War in 1985 and Doctors Without Borders in 1999 (1).

Doctors Against Forced Organ Harvesting (DAFOH) has been nominated for the 2016 Nobel Peace Prize for decade long efforts in raising awareness and informing the medical community and society about the unethical organ harvesting, with a particular focus on China (2). Forced organ harvesting, removal of organs from a donor without obtaining prior free and voluntary informed consent, is not only a crime against humanity, but a serious threat to medical science in general. The nomination of DAFOH is the second one related to forced organ harvesting in China, as human rights lawyers David Kilgour and David Matas were nominated in 2010 for their investigative work uncovering 41 500 unexplained organ transplants in China between 2000 and 2004 (3).

Increased information on the topic, including significant contributions from DAFOH members, has led to the publication of the book *State Organs – Transplant abuse in China* in 2012, which describes medical, ethical, legal, and political underpinnings of state-sanctioned organ harvesting from prisoners of conscience in China (4). The topic is almost beyond-imagination: living prisoners of conscience are systematically examined and killed on the operating table for their organs on demand (3,4). In 2012, DAFOH initiated a petition to the United Nations High Commissioner of Human Rights, gathering more than 2 million signatures worldwide within 3 years. Members of DAFOH were featured in documentaries on the topic of organ harvesting in China (“*Human Harvest*” and “*Hard to Believe*”). Moreover, DAFOH members regularly publish research articles addressing the transplant abuse (5). DAFOH is the only international medical organization that emphasizes prisoners of conscience as the major target group for forced organ harvesting in China (2). Since 2006, mounting evidence has suggested that prisoners of conscience are killed for their organs in China, with the brutally persecuted Buddhist practice, Falun Gong, being the primary target (see European Parliament Resolution and Workshop on Organ harvesting in China; 6,7).

## History of China's transplant abuse

Over 110 000 transplants are performed globally per year, 10% of which are estimated by the WHO to be from illegal organ trafficking (8). Illegal transplant activity has been documented all over the world, and although India, Pakistan, and Kosovo are frequently mentioned in this regard, the largest and most controversial is the exploitation of prisoners in China, which goes beyond any ethical standards.

China performs the second highest number of organ transplants in the world, approximately 10 000 annually, and has relied on organ procurement from prisoners since the 1984 national policy on the use of organs from executed prisoners (4,9,10) – a practice that has been unequivocally denounced by international declarations and organizations including the Nuremberg Code, the Helsinki Declaration, the Belmont report, World Medical Organization (WMA), World Health Organization (WHO), and the Transplantation Society (TTS) (11-16). The free donation rate was extremely low as China lacked a deceased organ donation system before 2010. In clear contrast to the approximately 120 000 total organs transplanted in China from 1977 to 2009, a total of 130 deceased organ donations were recorded in the same period. Initially, China denied the use of prisoner organs, but in 2005 Chinese officials admitted that more than 90% of these organs came from executed prisoners (4,9,10).

Further analysis of China’s transplant activity data uncovered glaring discrepancies – inordinate disparity between the number of executed prisoners and the number of transplants, publicly advertised organ wait times of only 2-4 weeks, and verifiable accounts of prescheduled transplants (3,4,9,10). The gap in numbers, the impossibility of each matching prisoner being executed on a needed date in combination with extremely short wait times strongly implied a large pool of prescreened organ sources that were executed and organ harvested “on-demand.” Comprehensive investigations suggested forced organ procurement from persecuted minority groups, including a large number of Falun Gong prisoners of conscience, which have been put to death on the basis of their spiritual belief. While in detention, they are systematically medically examined, blood tested, and screened – a costly practice that is unprecedented in China's prison system. Although they remain the largest persecuted group, there is evidence of similar fates for other minority groups – Uighur Muslims, Tibetans, and Christians (3,4). The prison system in China is tied to military hospitals, which are under the command of the People’s Liberation Army and have a unique political standing and autonomous status. Therefore, the regulatory changes in the civilian hospitals will not necessarily reflect the military ones, and both systems need to be reorganized in parallel (4,9,10).

In 2010, a pilot organ donation program was introduced and evolved into a national program and distribution system in 2013 – the China Organ Transplant Response System (COTRS) (9,10). However, the program does not completely conform to the international standards – significant financial compensation is provided, contrary to WHO Guiding Principles stating organ donation must be “unpaid and truly voluntary” (12). In December 2014, Chinese officials announced that only voluntarily donated organs could be used for transplantation. This was widely reported in the global media as a possible sign of improvement, but it was necessary to stop the practice completely. The announcement was not followed by any changes to organ donation laws or governmental regulations. Thus, a few questioned the credibility of the announcement and disclosed a semantic trick: prisoners are allowed to “voluntarily donate” organs (9).

## Making a change

Immediate and decisive action is needed from China to abolish the 1984 law on use of organs from executed prisoners, followed by a full ban, without any delay, in all hospitals, including military ones. The numbers of transplantations and executions should be disclosed and the Chinese transplant registries made public. Neither direct nor indirect forms of payment should be practiced to conform to the WHO Guiding Principles.

The international demand for transparency and scrutiny is crucial to the implementation of these recommendations. European Parliament in December 2013 issued a resolution, calling for an immediate end to forced organ procurement in China (6). House Resolutions 281 and 343 in the US Congress call for an end to the practice of organ procurement from prisoners, especially prisoners of conscience, in China and prosecution for those engaged in such practices (17). Israeli and Spanish parliaments enacted new organ transplant laws in 2008 and 2010 to deal with organ trade, halting the flow of Israeli and Spanish patients to China for organs (18). The journal publishers of transplantation research recognized the breaches of the ethical code stipulated by the Declaration of Helsinki, and Chinese authors have been denied publication rights by major scientific journals (19).

International sanctions, like the academic embargo, and dialogue with China have substantially contributed to China making steps in the right direction, such as the forming of national donation program in 2013 (9,10). However, there is no space for compromise – the credibility of China’s transplant medicine depends on clear regulations, which should prohibit the use of prisoner organs and provide transparency for the organ donation program.

Despite longstanding international condemnation, and repeated assurances from China of its plans to cease the practice, the organ procurement from prisoners continues today in China and even pulls the global population into the unethical practice by attracting transplant tourists. In December 2014, Chinese officials announced the intent to end organ harvesting from executed prisoners. The announcement, however, did not address organ harvesting from prisoners of conscience. Furthermore, statements in 2015 classified death-row prisoners as regular citizens who can donate their organs – a definition unique to China. Moreover, it in effect enables the use of organs from both death-row prisoners and prisoners of conscience (9). This concept is condemned by leading medical bodies – WMA, WHO, and TTS (11-13).

The need for action has now been especially emphasized following the publishing of a new in-depth report based on a meticulous examination of transplant programs of hundreds of hospitals, estimating the number of transplantations in China to be 60 000-100 000 per year. This figure is not only several times higher than the previously stated numbers, but also makes forced organ harvesting the only possible explanation for the estimate, while at the same time unavoidably widening its assumed scale dramatically (20).

There is a sincere hope that progressive, ethical, Chinese transplant professionals and government officials will emerge, embrace ethical standards, and recognize errors of the past, enabling China to take its place among the international transplantation community as a respected member. Until then, the international medical community should consistently demand a complete and immediate stop of the unethical practice of forced organ procurement. Transplant medicine might be a niche discipline in the wide field of medicine, but transplant abuse in China is a giant topic in medical ethics, challenging the entire medical profession and calling for the attention of all of us.

The Nobel Prize nomination is an acknowledgment of the work in directing the world's attention to the gross violations of medical transplant ethics in China – the only nation on earth that has systematically used its hospital system in coordination with the prison system to supply organs from non-consenting prisoners of conscience to fuel a lucrative transplant tourism industry. As medical community, we cannot let this abuse of transplant medicine continue unabated and must not give the impression of tacit acceptance. By direct or indirect involvement in initiatives aimed to stop this unethical practice, we undoubtedly condemn it and stand for our own ethical principles, making the world less of “a dangerous place to live in” because we do something about it.
